# Merging Remote Sensing Derived River Slope Datasets with High-Resolution Hydrofabrics for the United States

**DOI:** 10.1038/s41597-025-05941-6

**Published:** 2025-10-20

**Authors:** Yixian Chen, Sagy Cohen, Anupal Baruah, Dipsikha Devi, Supath Dhital, Dan Tian, Dinuke Munasinghe

**Affiliations:** Department of Geography and the Environment, The University of Alabama, Tuscaloosa, AL 35401 USA

**Keywords:** Hydrology, Natural hazards

## Abstract

The CONtiguous United States scale (CONUS) Flood Inundation Mapping Hydrofabric - ICESat-2 River Surface Slope (FIM HF IRIS) dataset integrates satellite-derived global IRIS river slopes for 117,357 spatially corresponding main-stream reaches within National Oceanic and Atmospheric Administration (NOAA) Office of Water Prediction operational FIM forecasting system (OWP HAND-FIM). A spatial joining approach was first developed to align FIM HF and IRIS reaches, addressing differences in reach flowline sources. Original FIM HF slopes had an average bias of 76 ± 168% relative to IRIS slopes. Applying to OWP HAND-FIM, FIM HF IRIS improved FIM accuracy by average 31 ± 25% (CSI) across eight flood events compared to the FIM HF slopes. Using a common attribute, IRIS data were transferred from FIM HF IRIS to the CONUS Next Generation Water Resources Modeling Framework Hydrofabric (NextGen HF), creating the NextGen HF IRIS dataset. Leveraging a common attribute, the resulting datasets enable using SWOT vector data within OWP HAND-FIM and NextGen. The spatial joining approach enabling integrating the hydrofabrics with other hydrologic datasets via flowlines is provided.

## Background & Summary

River slope (specifically water surface slope), also known as hydraulic gradient or flow gradient, refers to the slope of hydraulic grade line, which is the change in pressure head per unit distance^1–3^. Simply put, river slope is the difference in water surface elevation between an upstream and downstream point on a river, divided by the length of the reach^4–6^. It is a fundamental parameter in hydrologic and geomorphic modeling^5^, determining the transport and erosion capacity of rivers^7^. Furthermore, river slope is crucial for correcting WSE observations from satellite altimetry, thereby improving the accuracy of water level time series^8,9^.

Under uniform flow conditions, the bed slope, water surface slope, and slope of the energy grade line are considered equivalent^10^. In addition, obtaining accurate bed slope measurements, especially at large scales, is very challenging, making water surface slope a commonly used substitute. River slope is widely used as a key input for calculating flow velocity and river discharge based on Manning’s equation^11–14^.

Flood Inundation Mapping hydrofabric (FIM HF) is developed at the United States (US) scale using river slope data as an important input. There hydrofabric is an underlying set of data used to create a digital representation of a hydrologic model^15^. FIM HF is the product of the National Oceanic and Atmospheric Administration (NOAA) Office of Water Prediction (OWP) operational FIM forecasting system, which employs the Height Above Nearest Drainage (HAND) approach to generate flood maps (referred to as OWP HAND-FIM). FIM HF is generated including stream network, catchments, HAND grids, synthetic rating curves (SRCs), and cross-walk table, defined as the dataset required to make an inundation map from discharges^16^. HAND is defined as the height of each grid cell with respect to the nearest stream cell it drains. The SRC reflects the relationship between discharge and the average HAND stage at the reach scale. In OWP HAND-FIM, SRCs are used to convert riverine discharge to a stage value to generate inundation extent for a given reach^15,16^. The OWP HAND-FIM SRCs are calculated using Manning’s equation (Eq. 1) and are thus sensitive to reach-averaged slope values.

1 $$Q={VA}=\frac{{k}_{n}}{n}A{R}^{\frac{2}{3}}{S}^{\frac{1}{2}}$$

Where *Q* is the discharge, *V* and *A* is respectively the cross-sectional average velocity and area, *R* is the hydraulic radius, *S* is the river slope, *k*_*n*_ = 1 for international system of units (i.e. *R* in meters and *V* in m/s) and *k*_*n*_ = 1.486 for English units (i.e. *R* in feet and *V* in ft/s), and *n* is the Manning roughness coefficient.

Operationally, the National Water Model (NWM) forecast streamflow predictions are used in conjunction with the OWP HAND-FIM to derive forecast flood inundation maps^16^. The NWM hydrofabric has undergone considerable development as part of the OWP Next Generation Water Resource Modeling Framework (NextGen)^17,18^. The NextGen framework will be used in version 4.0 of the NWM^19^. The river slope attribute of the NextGen HF is important primarily for the NextGen flow routing modules (e.g. T-Route)^20,21^.

There may be issues with the river slope attribute for both hydrofabrics. FIM HF initially used the 10 m National Hydrography Dataset Plus High Resolution (NHDPlusHR) DEM dataset^22^, derived from the 3D Elevation Program (3DEP)^23^, to derive the river slopes^16,24^. However, 3DEP continues to advance using the latest elevation sources, such as LiDAR, and expanding its coverage (https://www.usgs.gov/3d-elevation-program). It was demonstrated that using the 3DEP 10 m DEM significantly improved the quality of FIM extents in 80% of the catchments analyzed when compared to the NHDPlusHR 10 m DEM, with the mean catchment-scale critical success index (CSI) increasing from 0.55 to 0.63^24^. That indicates the higher quality of the 3DEP DEM than the NHDPlusHR DEM. FIM HF switched to the 3DEP DEM starting with version 4.0.10.0 (released on October 4, 2022; https://github.com/NOAA-OWP/inundation-mapping/blob/dev/docs/CHANGELOG.md). Despite this, in FIM HF, a default minimum river slope of 0.001 mm/mm (~0.06 degrees) is often assigned to streams to avoid zero values, as a zero slope would make discharge calculations impractical for the construction of SRCs. However, this default value can be problematic, as it is quite high (median river slope in North America was estimated to be 0.014 degrees^25^) and likely deviate considerably for the true slope. Also, NextGen HF derives river slopes from 3DEP 10 m DEM^26^.

The Ice, Cloud, and Land Elevation Satellite-2 (ICESat-2), equipped with the Advanced Topographic Laser Altimeter System (ATLAS) (https://nsidc.org/data/icesat-2), provides an alternative source of elevation data. Among the 22 products generated by ICESat-2 ATLAS, ATL13 specifically measures inland water surface height and appears to offer higher precision than the 3DEP 10 m DEM, with an average root mean squared error (RMSE) of 0.24 ± 0.10 m^27–29^, compared to an average RMSE of 0.75 ± 0.67 m for the 3DEP 10 m DEM^30–32^. Furthermore, ICESat-2 was launched in 2018 and has a revisit period of 91 days (https://icesat-2.gsfc.nasa.gov/articles/counting-nasas-icesat-2), offering recent data with higher temporal resolution compared to the 3DEP DEM^24,33,34^.

Given its advantages, several studies have used ICESat-2 data to derive river slopes^6,8,35,36^. The global reach-scale ICESat-2 River Surface Slope (IRIS) v1 dataset was introduced recently^35^, which includes average and extreme water surface slopes derived from ICESat-2 observations between October 2018 and August 2022 for 121,583 reaches in the Surface Water Ocean Topography (SWOT) Mission River Database (SWORD) stream network^25^. Studies show that IRIS slopes are more accurate than the other river slope datasets, e.g. SWORD and GloRS^5,6,37^. Importantly, the SWOT vector dataset (https://podaac.jpl.nasa.gov/dataset/SWOT_L2_HR_RiverSP_2.0), derived from the SWOT mission^38^ and containing river slope data assigned to SWORD reaches, can be cross-validated with IRIS data once large-scale SWOT observations become available. The latest IRIS dataset (v3.0) significantly expands slope calculations for more SWORD reaches (138,065 globally) using updated ICESat-2 observations between October 2018 and August 2024^39^, providing large potential in improving river slopes in the FIM HF and NextGen HF.

In this paper, we explore the utilization of satellite-derived IRIS river slopes within the high-resolution hydrofabrics FIM HF and NextGen HF. This is motivated by potential improvements in river slope accuracy within hydrofabrics, which can, in turn, improve flood inundation mapping, an increasingly urgent concern under climate change, as well as hydrological predictions. Moreover, the availability of temporally dynamic SWOT-measured river slopes can potentially transform the parameterization of river slopes within large-scale hydrological applications. Additional datasets are also being integrated with SWORD, such as the Multi-Error-Removed Improved Terrain (MERIT)-Basins dataset^40^. While realizing these integrations is beyond the scope of this paper, the tools and datasets presented herein are critical elements of connecting data from multiple sources and enabling their reciprocal use. The first aspect of integrating satellite-derived river slopes within hydrofabrics is linking their stream networks. The two main challenges associated with this are spatial mismatch and river segmentation. The former refers to the spatial misalignment between the two vector flowline layers. The latter refers to the topology of each stream network. Here, we developed a dedicated spatial joining procedure to link SWORD flowlines with FIM HF and NextGen HF flowlines. We analyze the accuracy of IRIS river slopes and their capacity to improve FIM accuracy by comparing FIMs derived using the IRIS river slopes, original FIM HF river slopes, and NextGen HF slopes. The resulting datasets of FIM HF and NextGen HF, incorporating IRIS river slopes, are presented, which include a link (common ID) enabling the incorporation of SWOT observations.

## Methods

The IRIS and FIM HF use flowlines from different sources, a spatially joining approach was developed to generate FIM HF IRIS. Then based on common attributes between the FIM HF and the NextGen HF, the IRIS river slopes from FIM HF IRIS were transferred to the NextGen HF, generating the NextGen HF IRIS. The required input data are listed in Table 1.

**Table 1:** Input data for FIM HF IRIS and NextGen HF IRIS datasets.

Dataset	Variable	Description
Office of Water Prediction Height Above Nearest Drainage Flood Inundation Mapping Hydrofabric (FIM HF)^15^		
	HydroID	Unique identifier for FIM HF stream flowline
	NextDownID	HydroID of the immediate downstream flowline
	From_Node	Upstream node ID
	To_Node	Downstream node ID
	feature_id	Common identifier value for cross-walked NWM flowline
	Stream network (flowline)	Reach centerline shapefile geometry (used to carry the integrated IRIS slopes)
	LengthKm	Length of HydroID flowline (unit: km)
Next Generation Reference Hydrofabric (NextGen HF)^26^		
	id	Unique identifier for NextGen reference HF flowpath
	flowpath (flowline)	Reach centerline shapefile geometry (used to carry the IRIS slopes)
ICESat-2 River Surface Slope (IRIS)^35,43^		
	reach_id	The SWORD reach identifier
	avg_combined_slope	Average ICESat-2 combined slope for the reach (unit: mm/km)
SWOT Mission River Database (SWORD)^25,51^		
	reach_id	The SWORD reach identifier (used as key to join the IRIS to FIM HF)
	centerline (flowline)	Reach centerline shapefile geometry (used to find the spatially corresponding FIM HF flowlines)
	slope	Reach average slope (unit: m/km)

After integrating IRIS river slopes into both hydrofabrics, the accuracy of IRIS river slopes relative to the original FIM HF and NextGen HF slopes was evaluated. To do this, FIMserv, a toolset for the calculation of the OWP HAND-FIM^41^, was used to generate flood maps with both the original FIM HF river slopes and slopes from IRIS and NextGen HF. The maps were then assessed using FIMeval (Flood Inundation Mapping Predictions Evaluation Framework), an automated tool for evaluating flood maps using diverse benchmark FIMs^42^, including remote sensing (RS) imagery-derived benchmarks.

### Input datasets

#### FIM Hydrofabric

Since version 4.0.10.0, the FIM HF is derived by OWP from the processed 3DEP DEM and linked to the NWM (v2.1) stream network through a reach identifier attribute (*feature_id*; Table 1). FIM HF flowlines length were set to a maximum of 1.5 km to improve SRC and FIM skills^16^. The attribute *HydroID* is the unique identifier of FIM HF flowlines, along with a *NextDownID*, which is the HydroID of the downstream flowline, which can combine and reflect the flow direction and connectivity of FIM HF reaches^16^. The FIM HF original river slope was calculated as the depression between elevations at both upstream and downstream of the reach, sampled from the processed 3DEP DEM^23^, divided by the reach length^16^.

#### NextGen hydrofabric

The NextGen HF utilizes the National Hydrography Dataset Plus version 2 (NHDPlusV2) flowlines as the fundamental flow network, which consists predominantly of river and artificial path features. River slope data of the latest NextGen HF (v2.2) were computed for the flow network using the 3DEP 10 m DEM. In NextGen HF v2.2, the flowline’s unique identifier is the *id* attribute (Table 1).

#### IRIS dataset

The global reach-scale ICESat-2 River Surface Slope (IRIS) dataset (v2.6) comprises of minimum, average and maximum water surface slopes derived from ICESat-2 observations between October 2018 and May 2024, as new attributes to the 180,939 reaches from the SWORD dataset^35,43^. SWORD provides a unique identifier *reach_id* for each IRIS record (Table 1). IRIS river slopes were validated at 815 reaches in Germany, France, Hungary, and the United States with a median absolute error (MAE) of 23 mm/km^8^, as well as for eight rivers in Poland with an average RMSE of 54 mm/km^6^, both compared to gauge data. The IRIS river slopes are obtained through combining the slopes of rivers across pairs of ICESat-2 LiDAR beams and along individual beams, to maximize spatial and temporal coverage^35^. In addition, the IRIS dataset includes other attributes such as the minimum, maximum, and standard deviation of river slopes during the observation period for each reach.

SWORD also serves as the river network database for SWOT river vector products, which provides water surface elevation, width, slope, and estimated discharge^25,44^. Therefore, by referencing SWORD as a common database, the SWOT river vector products, expected to have high accuracy^6,45^, can be linked.

### Methodology

#### Spatial joining procedure

The FIM HF (v4.5.2.11) flowlines and the IRIS (v2.6) flowlines, which (the latter) use SWORD (v16) flowlines, originate from different sources and hence do not match spatially and topologically. Additionally, SWORD contains medium to large rivers (minimum width of about 30 m), whereas FIM HF also includes small rivers. A spatial joining process was therefore developed to link FIM HF and SWORD flowlines. Since IRIS only uses the reaches in the SWORD dataset that passed the *type* flag filter (i.e. types “*river*” and “*lake on river*”, excluding “*lake off river*”, “*dam or waterfall*”, etc.), we used the original SWORD flowlines to ensure that the future SWOT dataset (relying on SWORD reaches) can be fully linked to FIM HF.

**Fig. 1:**
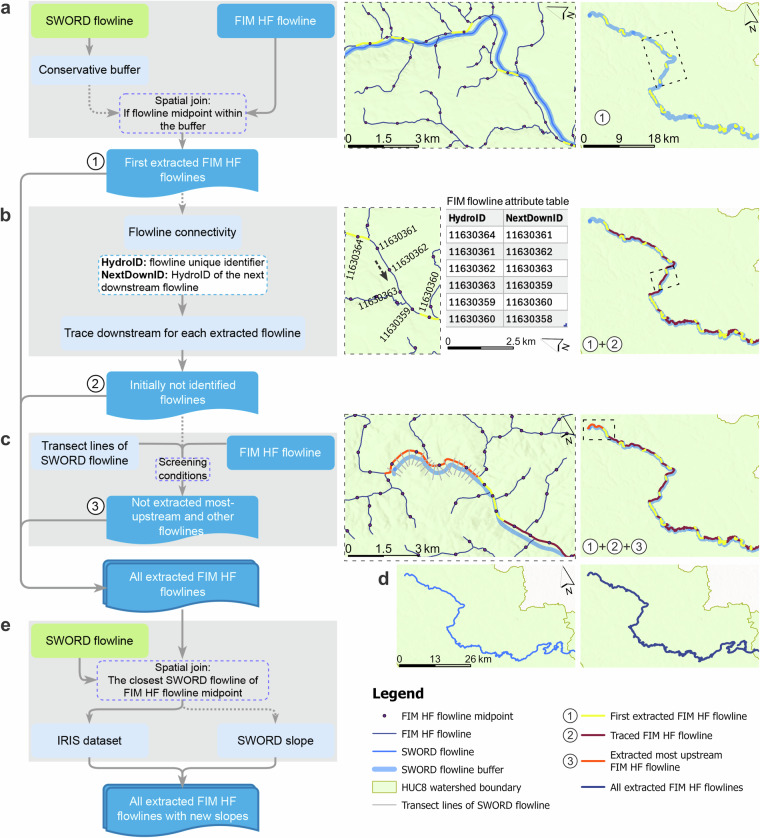
Flowchart of identifying and spatially joining FIM HF flowlines with corresponding SWORD flowlines. Middle-column figures enlarge the dashed-line box areas in the 3rd column.

FIM HF is stored at the 8-digit Hydrologic Unit Code (HUC8) watershed scale, which divides the US into about 2400 HUC8 watersheds^16^. Due to the significantly less available data of both hydrofabrics and SWORD in the non-contiguous states and territories, the spatial joining and generation of FIM HF IRIS and NextGen HF IRIS are focused on the CONUS scale. The spatial joining of FIM HF and SWORD flowlines was conducted at the HUC8 watershed scale, of which the detailed procedures are presented in Fig. 1. First, a buffer with a conservative 100 m distance was created around the SWORD flowlines. This buffer enabled a spatial join with the midpoints (those located within the buffer) of the FIM HF flowlines, ensuring that basically half of the corresponding FIM HF flowline was located within the buffer, effectively excluding irrelevant tributaries (Fig. 1a). The flowlines with midpoints intersecting the SWORD buffer were extracted (Fig. 1a). This approach ensured that most of the FIM HF flowlines spatially aligned with SWORD reaches can be identified effectively (Fig. 1a).

Once the intersecting FIM HF flowlines were identified, their connectivity was traced downstream using the *HydroID* field (unique FIM HF flowline identifier) and the *NextDownID* field (HydroID of immediate downstream flowline) in the attribute table (Fig. 1b, Table 1). For each identified flowline, downstream tracing was conducted until the first identified flowline was reached. For the most downstream identified flowline, this tracing was conducted until the most downstream flowline in the watershed was reached. This process extracted the FIM HF flowlines not initially identified through the buffer but spatially corresponding to SWORD flowlines (Fig. 1b).

The above processes could not identify the most upstream and some other FIM HF flowlines that corresponded to SWORD flowlines (Fig. 1c). To address this, transect lines, perpendicular to and evenly distributed along the SWORD flowlines, were generated. Here we used transect lines at a length of 600 m perpendicular to the SWORD flowlines at each 200 m (Fig. 1c). If the transect lines intersected with any FIM HF flowlines already identified through the two steps above (Fig. 1a,b), they were eliminated (Fig. 1c). For the remaining transect lines, if FIM HF flowlines intersected with three transect lines or intersected with at least one transect line and the flowline stream order (attribute *order_*) ≥ 2, they were extracted as corresponding to the SWORD flowlines (Fig. 1c). Through our iterative tests, we found that with these three steps, almost all the corresponding FIM HF flowlines can be accurately extracted. Then, all the identified flowlines were combined into one layer (Fig. 1d).

In the final step, the midpoint of each FIM HF flowline was used to locate the nearest SWORD streamline, ensuring alignment over at least half the flowline. After that, the *reach_id* field from SWORD streamline was appended to the corresponding FIM HF flowline in the attribute table. Based on this common field (Table 1), IRIS data were joined to FIM HF (Fig. 1e). All above spatial joining steps were performed in ArcGIS Pro (v3.3.2). A Jupyter Notebook script was developed to automate this process at the HUC8 watershed scale for the entire hydrofabric. The spatial joining approach developed here can also be applied to connect stream networks from other datasets. It has then been converted into a robust Python Jupyter Notebook script, requiring only the paths and names of the flowline layers and their unique identifier fields. Additional parameters, such as buffer distance (Fig. 1a), perpendicular line spacing and length, and filter expressions (Fig. 1c), can be specified to customize the join process. The Python script is publicly available on GitHub: https://github.com/YixianChen-234/RiverJoin.

If a feature (stream reach) in the joined FIM HF contains no available IRIS data, the slope value from the SWORD is used (Fig. 1e). The SWORD slope attribute was calculated using MERIT Hydro elevations and linear regression^25^ and has also been found to have an overall good accuracy^6^. The source of slopes in the resulting datasets is indicated in the *slope_source_iris_sword* column (Table 2).

**Table 2:** Contents of FIM HF IRIS and NextGen HF IRIS datasets.

Variable Name	Unit	Description
**FIM HF IRIS dataset v1.0**		
HydroID	—	Unique identifier for FIM HF stream segments
From_Node	—	Original FIM HF upstream node ID
To_Node	—	Original FIM HF downstream node ID (used in combination with HydroID and From_Node to ensure exact correspondence with FIM HF flowlines when using the FIM HF IRIS dataset)
reach_id	—	The SWORD reach identifier (can be used to join the IRIS and SWOT to FIM HF)
slope_iris_sword	mm/mm	The IRIS slope joined to the corresponding FIM HF flowline, while the SWORD slopes were used for the flowlines where IRIS slopes were unavailable
slope_source_iris_sword	—	Value indicating the slope of *slope_iris_sword* is from IRIS (1) or SWORD (2)
**NextGen HF IRIS dataset v1.0**		
id	—	Unique identifier for NextGen HF flowline (flowpath)
reach_id	—	The SWORD reach identifier (can be used to join the IRIS and SWOT to NextGen HF)
slope_iris_sword	mm/mm	The IRIS slope joined to the corresponding NextGen HF flowline, while the SWORD slopes were used for the flowlines where IRIS slopes were unavailable
slope_source_iris_sword	—	Value indicating the slope of *slope_iris_sword* was from IRIS (1) or SWORD (2)

#### Joining IRIS slope to NextGen HF flowlines

To incorporate the IRIS river slopes into the NextGen HF, we leveraged the FIM HF common ID with the NextGen HF. FIM HF flowlines already linked with IRIS data were therefore joined with the NextGen HF reference flowlines (v2.2), based on the common fields, i.e., *id* in NextGen HF and *feature_id* in FIM HF (Table 1), both being original unique identifiers for NHDPlusV2 flowlines^16,26^. However, because each stream in FIM HF is split into equidistant reaches not to exceed 1.5 km^16^, many NextGen HF flowlines correspond to multiple FIM HF reaches (one *id* to many *feature_id*s). To link the most relevant IRIS river slope to NextGen HF, the mode *reach_id* of the FIM HF flowlines is assigned to a corresponding NextGen HF flowline. If there is no mode *reach_id* available, then the longest FIM HF reach (determined by *LengthKm* attribute) is used (Table 1). The FIM HF flowlines having a one-to-one relation with NextGen HF flowlines are joined directly through the common fields. After this, the IRIS river slope can be linked from FIM HF IRIS to NextGen HF flowlines using the field *reach_id*.

## Data Records

The CONUS scale FIM HF IRIS v1.0 and NextGen HF IRIS v1.0 datasets are available at Zenodo and HydroShare^46,47^. Each of the datasets is stored in a single CSV file, which respectively contains the variables listed in Table 2. The FIM HF IRIS can be used in OWP HAND-FIM via the common *HydroID* field, in combination with the *From_Node* and *To_Node* fields to ensure exact match. The NextGen HF IRIS can be joined to NextGen HF via the *id* field. Additionally, feature classes of FIM HF IRIS and NextGen HF IRIS flowlines stored in geodatabases (.gdb) and geopackages (.gpkg), which include the incorporated slope data in their attribute tables, have also been uploaded^46,47^. This enables users to visually inspect, map, and better utilize the new slope data. The SWOT Level 2 River Single-Pass Vector Data Product—featuring variables such as water surface elevation, river slope, and river width, etc., derived from the latest SWOT High Rate Pixel Cloud product^44^—can be integrated into these two datasets (FIM HF IRIS and NextGen HF IRIS) using the common *reach_id* field (Table 2).

**Fig. 2:**
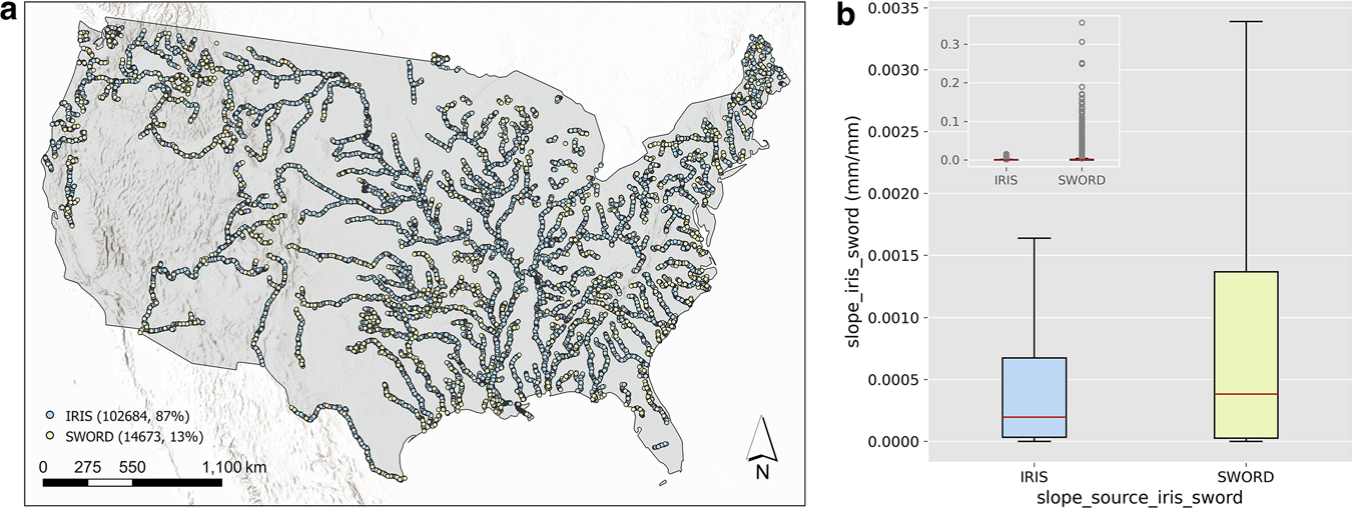
Source of *slope_iris_sword* (**a**) and comparison of IRIS and SWORD slopes (**b**) in the FIM HF IRIS dataset. The points in (**a**) are the midpoints of FIM HF flowlines; the inset in (**b**) shows the raw boxplots with outliers.

As stated, the IRIS dataset does not provide slope values for all SWORD reaches which is addressed here by using the SWORD original slope values for those reaches. The final incorporated slope variable for the two hydrofabrics was named *slope_iris_sword* (Table 2). In the FIM HF IRIS, 87% of reaches have a slope value from the IRIS dataset while 13% are from SWORD (Fig. 2). The source of the slope dataset in the datasets is recorded in the *slope_source_iris_sword* attribute (Table 2). For the other reaches not included in SWORD, users can utilize the original river slopes of the FIM HF (*S0* attribute) and NextGen HF (*So* attribute). Links for accessing the original FIM HF and NextGen HF are provided in the Code Availability section.

**Fig. 3:**
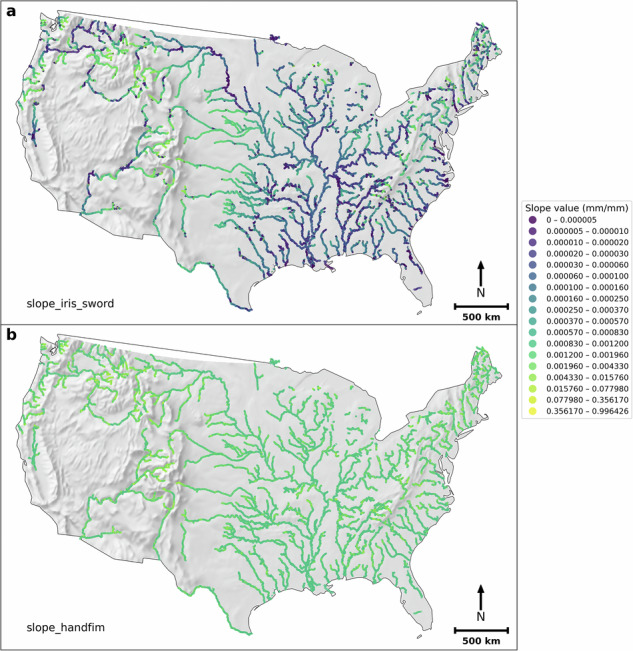
IRIS (and SWORD) slopes (*slope_iris_sword*) (**a**) and the original FIM HF slopes (**b**) in FIM HF IRIS.

**Fig. 4:**
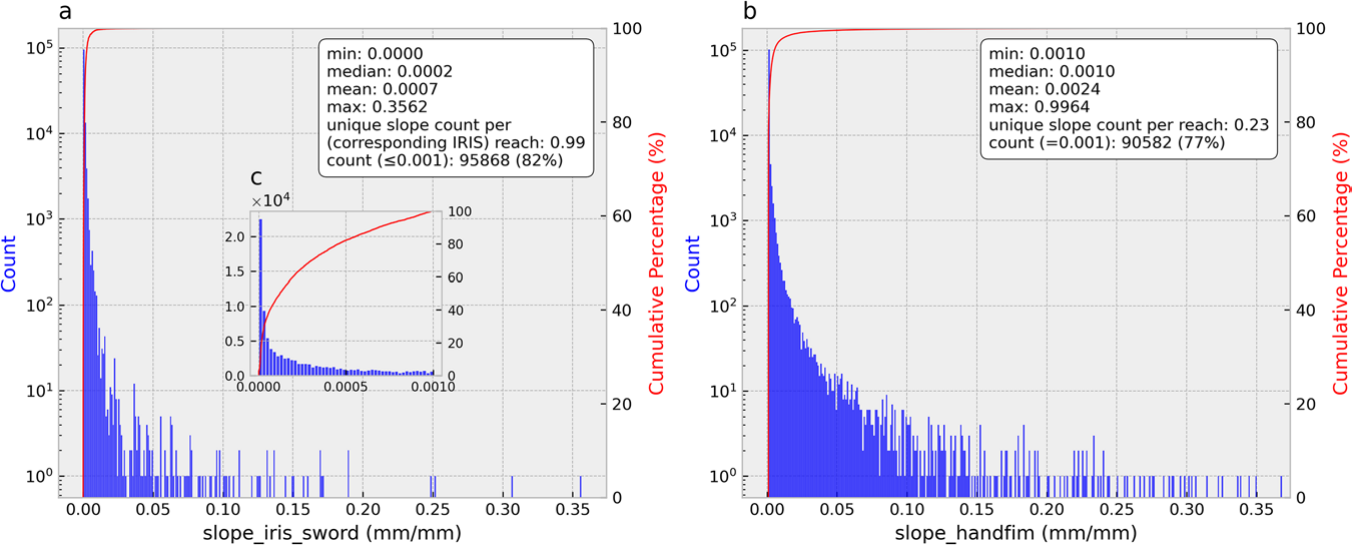
Distributions and basic statistics of *slope_iris_sword* (**a**) and *slope_handfim* (**b**) in FIM HF IRIS. (**c**) The distribution of *slope_iris_sword* ≤ 0.001. The unique slope count per reach was calculated as the number of unique slope values divided by the number of reaches, indicating slope variability. The *slope_handfim* x-axis was truncated to match that of *slope_iris_sword* for direct comparison.

There are 117,357 extracted FIM HF reaches that have a corresponding SWORD flowline. Figure 3 shows the corresponding IRIS (and SWORD) slopes (*slope_iris_sword*) and the original FIM HF slopes (*slope_handfim*). It can be observed that *slope_iris_sword* has more richness (unique values, Fig. 4) than FIM HF slopes (Fig. 3). About 77% of the FIM HF main-streams just have a river slope of 0.001 mm/mm (Fig. 4b), the default minimum river slope in FIM HF. Furthermore, *slope_iris_sword* slopes have smaller mean values than the *slope_handfim*, because the 0.001 value in a large number of reaches corresponds to a relatively high slope (North America median slope in SWORD is 0.00025^25^). In addition, the original river slopes in FIM HF, other than 0.001, are generally higher than those of *slope_iris_sword* (Fig. 4).

**Fig. 5:**
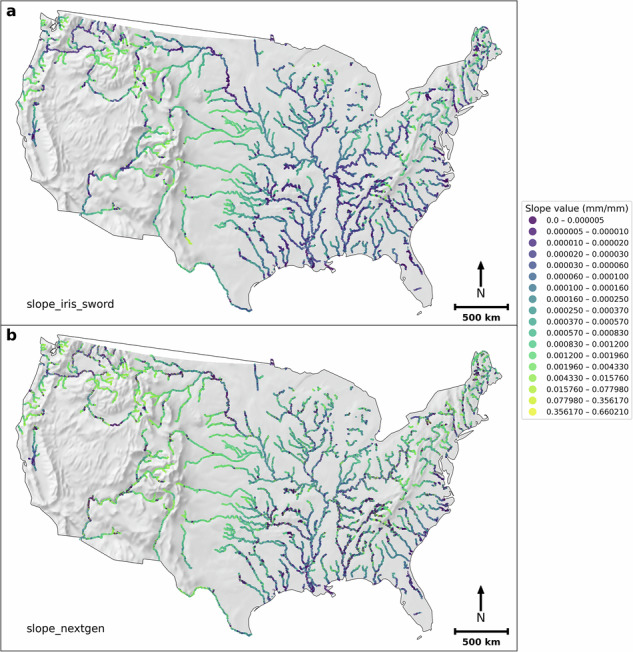
*slope_iris_sword* (**a**) and the original NextGen HF slopes (**b**) in NextGen HF IRIS.

**Fig. 6:**
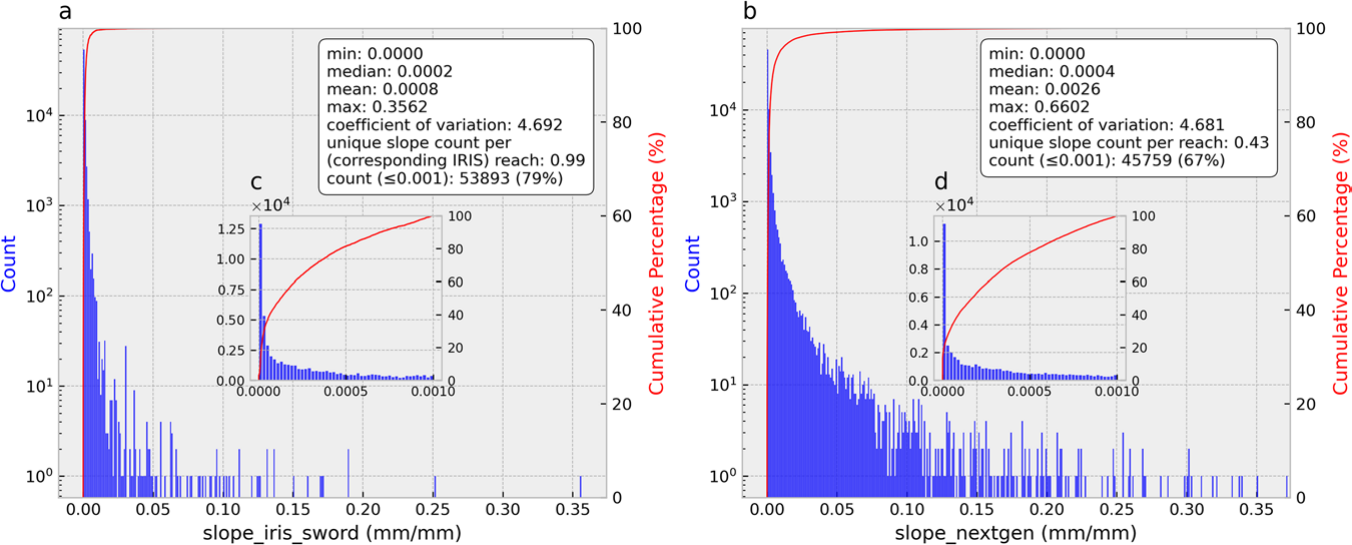
Distributions and basic statistics of *slope_iris_sword* (**a**) and *slope_nextgen* (**b**) in NextGen HF IRIS. (**c,d**) show distributions for slope values ≤ 0.001. The *slope_nextgen* x-axis was truncated to match that of *slope_iris_sword* for direct distribution comparison.

Figure 5 shows *slope_iris_sword* and the original NextGen HF river slopes (*slope_nextgen*) in the NextGen HF IRIS. There are 68,275 NextGen HF reaches having corresponding reaches in the FIM HF IRIS. The *slope_iris_sword* slopes in NextGen HF IRIS have more variability than *slope_nextgen* (higher coefficient of variation and unique slope count per reach, Fig. 6). Moreover, *slope_iris_sword* slopes are generally lower than *slope_nextgen* (Fig. 6), but the differences are smaller than those between *slope_iris_sword* and *slope_handfim* in FIM HF IRIS (Figs. 3, 4). These two points imply that the original NextGen HF slopes may be more accurate than the FIM HF river slopes.

## Technical Validation

The approach used to derive IRIS data was originally validated at 815 reaches in Europe and the U.S.^8^ with a MAE of 23 mm/km compared to gauge data. Further validation indicated that IRIS has an RMSE of 54 mm/km for 8 rivers in Poland when compared with the *in-situ* river slopes between gauging stations, outperforming other commonly used sources of (estimating) river slope, such as GloRS, 1 km LiDAR, and RiverProfileApp^6^. SWORD river slopes were also found to have good correspondence to *in situ* observations, with an RMSE of 71 mm/km^6,8^. More validation of the IRIS dataset can be found in the data descriptor paper of IRIS^35^.

**Fig. 7:**
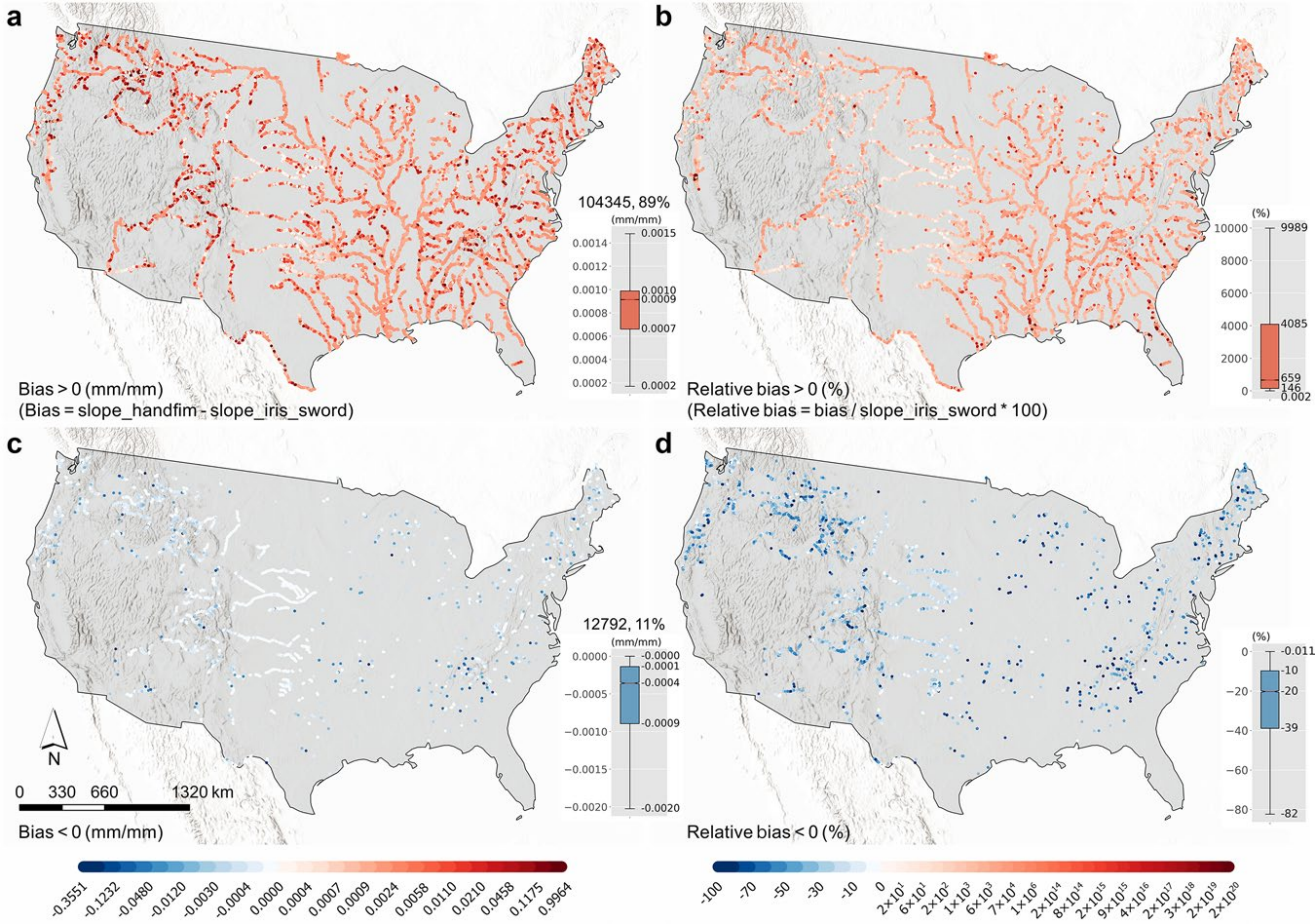
Bias and relative bias between *slope_iris_sword* and FIM HF slopes in the FIM HF IRIS dataset. The boxplots display the distribution of the biases. The numbers above the boxplots in (**a,c**) represent the count and percentage of reaches in FIM HF IRIS where the original slopes are either greater or smaller than the corresponding *slope_iris_sword* slopes. Note that the apparent duplication of reaches between the positive and negative (relative) bias plots is due to display scale, not actual duplication.

Although the CONUS-scale validation of IRIS and FIM HF slopes with gauged slopes is not feasible, the overall superiority of *slope_iris_sword* and the effectiveness of the spatial joining can be inferred from Fig. 3 and the following analyses. Figure 7 shows the bias and relative bias between the FIM HF slopes and IRIS. Most of the reaches (89%) have a FIM HF slope greater than the corresponding IRIS (Fig. 7a,b), which may be because, on the one hand, IRIS is a long-term aggregation using median from October 2018 to May 2024^35,43^ (also therefore able to better represent overall river steepness), but FIM HF slopes were derived from the partially older and single-observed 3DEP DEM dataset^23^ (https://www.usgs.gov/3d-elevation-program). On the other hand, there are 77% FIM HF reaches (Fig. 4b) having the default river slope (0.001 mm/mm), which should be greater than most of the corresponding IRIS slopes, given that there are 82% reaches having a river slope ≤ 0.001 mm/mm (Fig. 4a). These suggest the highly potentially higher accuracy of IRIS than FIM HF slopes. Furthermore, the *slope_iris_sword*s better capture the steeper river slopes in the Rocky Mountains and the Appalachians than FIM HF slopes (Fig. 7c,d), as normally in the steeper mountainous areas the river slopes also tend to be larger^5^, potentially indicating the high accuracy of *slope_iris_sword*.

**Fig. 8:**
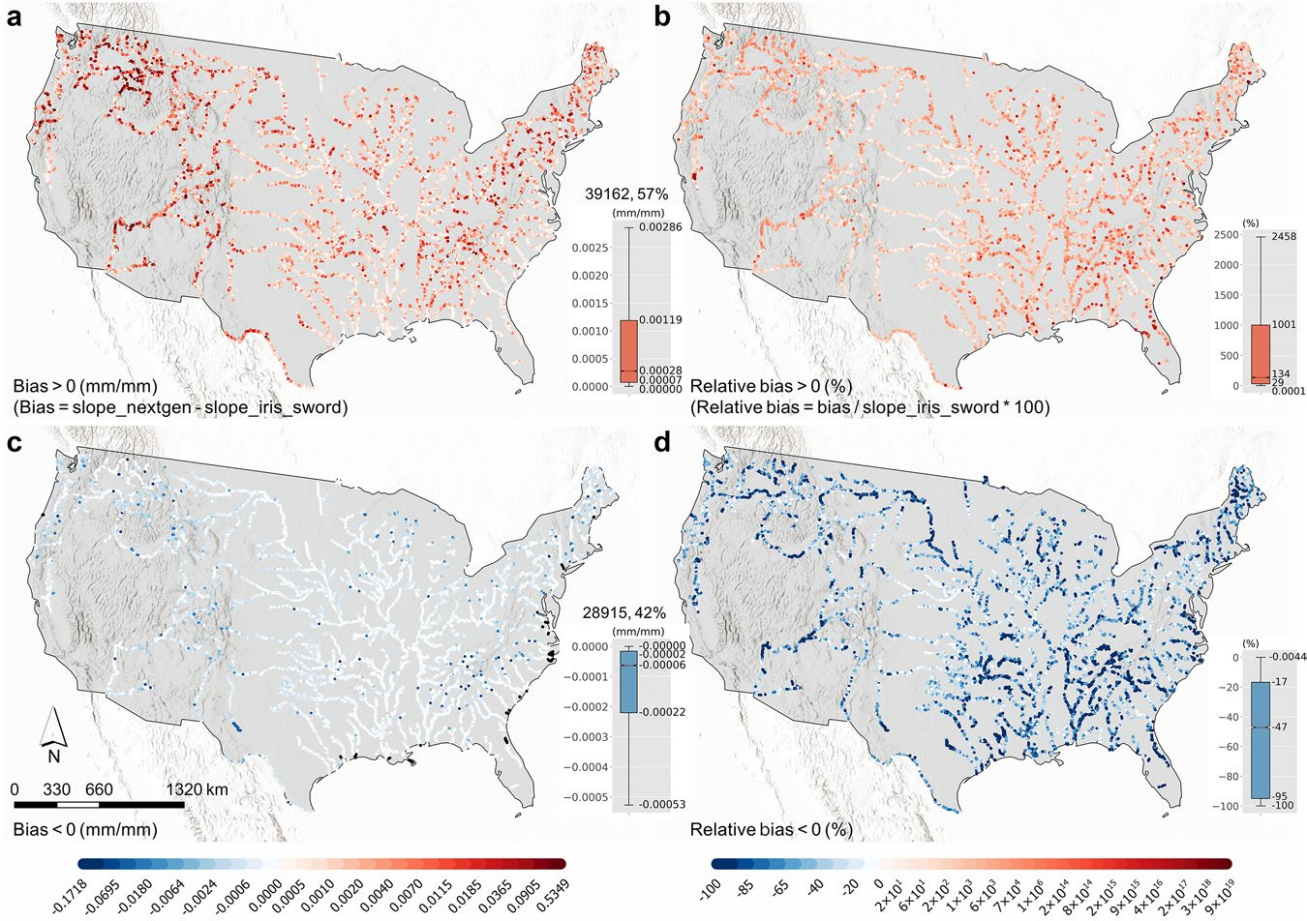
Bias and relative bias between *slope_iris_sword* and NextGen HF slopes in the NextGen HF IRIS dataset.

Figure 8 shows the bias and relative bias between the *slope_iris_sword* slopes and NextGen HF slopes. About 57% NextGen slopes are greater than *slope_iris_sword* slopes, which suggests that NextGen HF slopes have more variations than FIM HF slopes (also cf. the unique slope counts per reach in Figs. 4b, 6b) and may have higher accuracy than FIM HF slopes as well, especially also considering that NextGen HF slopes were calculated from more accurate 3DEP 10 m DEM^24^.

**Fig. 9:**
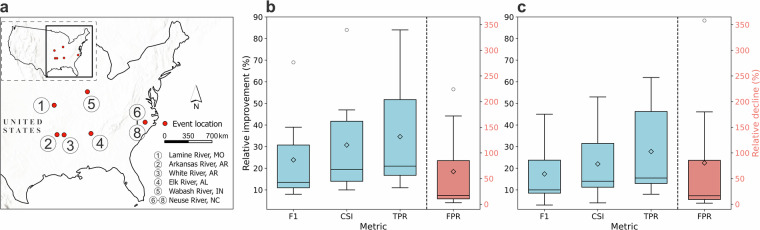
Distribution of evaluation sites where high-precision flood maps obtained from remote sensing imagery are available (**a**) and the box plots showing overall improvements in FIM evaluation metrics using *slope_iris_sword* (**b**) and *slope_nextgen* (**c**) relative to *slope_handfim*. F1, F1 score; CSI, critical success index; TPR, true positive rate; FPR, false positive rate.

To assess the impact of *slope_iris_sword* on flood inundation predictions, we generated flood maps in areas where high-precision flood maps derived from remote sensing (RS) imagery are available^48,49^. The flood inundation predictions were generated using the OWP HAND-FIM model with both *slope_iris_sword* and the original *slope_handfim* values. They were then evaluated, using FIMeval^42^, against RS flood maps for 8 flood events in 6 locations between 2016 and 2018 (Table 3, Fig. 9a). Six of the eight RS flood maps were validated against NOAA Emergency Response (ER) Imagery, with an overall accuracy of 98%, an omission error of 2%, and a commission error of 3%^48,50^. ER Imagery is very high-resolution airborne imagery (between 30 and 50 cm ground sampling distance between pixels) acquired by the NOAA Remote Sensing Division during major flood events in the U.S. to support NOAA’s homeland security and emergency response requirements. For the other two of the eight tested flood events, ER Imagery-based flood maps were used as benchmarks^49^. Multiple machine learning algorithms were applied to the raw imagery to generate initial flood maps. The map with the highest classification accuracy was then manually refined by an expert GIS analyst, with extensive experience in flood mapping, using raw ER imagery for detailed ground-level corrections.

**Table 3:** Information of the tested flood events and the remote sensing (RS) imagery used to derive benchmark flood maps.

Event No.	Location	Date of flood event (yyyy/mm/dd)	RS imagery used	Resolution of RS imagery (meter)
①	Cooper, Missouri	2017/05/03	Sentinel 1	10
②	Perry, Arkansas	2016/01/03	NOAA Emergency Response Imagery, Midwest US Flooding (2015)	~0.5
③	Prairie, Arkansas	2016/01/03	NOAA Emergency Response Imagery, Midwest US Flooding (2015)	~0.5
④	Limestone, Alabama	2018/02/11	Sentinel 1	10
⑤	Tippecanoe, Indiana	2017/05/06	Sentinel 1	10
⑥	Wayne, North Carolina	2016/10/09	PlanetScope Scene	3
⑦	Wayne, North Carolina	2016/10/14	PlanetScope Scene	3
⑧	Wayne, North Carolina	2016/10/15	PlanetScope Scene	3

Note: RS imagery acquired as close as possible to the same time on the same day as the flood events was used.

**Fig. 10:**
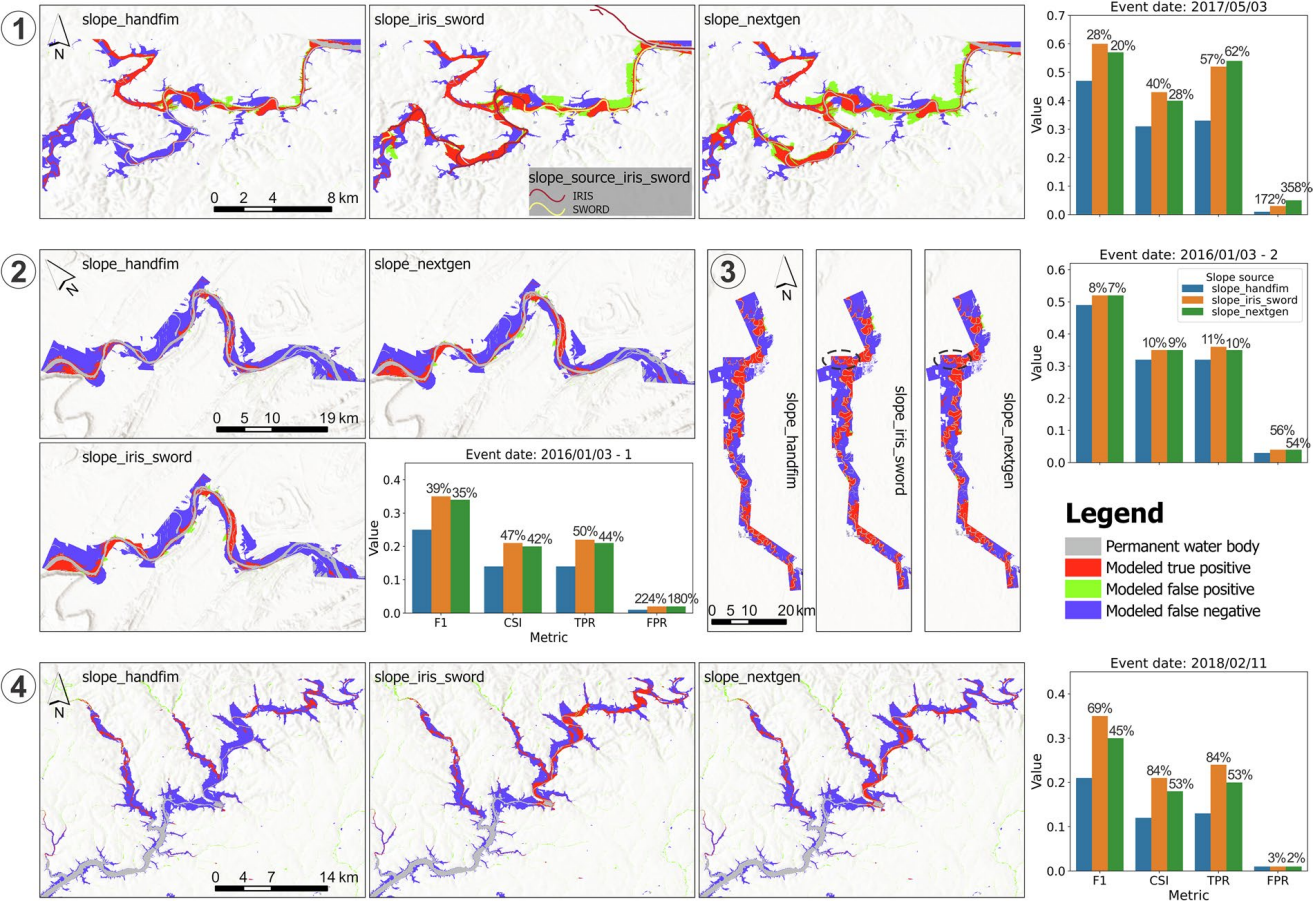
Comparison of flood maps based on OWP HAND-FIM using different slope sources and the evaluation results against benchmark RS FIM for case events 1–4 (Fig. 9a). Slope source is indicated in the upper left of each flood map; source of *slope_iris_sword* is only indicated for event 1 by flowline color, for the other events all slopes are from IRIS; evaluation results were presented as bar plots. The numbers above the bars indicate the relative difference of the metric between using *slope_iris_sword*, *slope_nextgen* and original FIM HF slopes. Dash-line circles highlight key FIM improvements relative to the original slopes for event 3, due to the minor improvements.

**Fig. 11:**
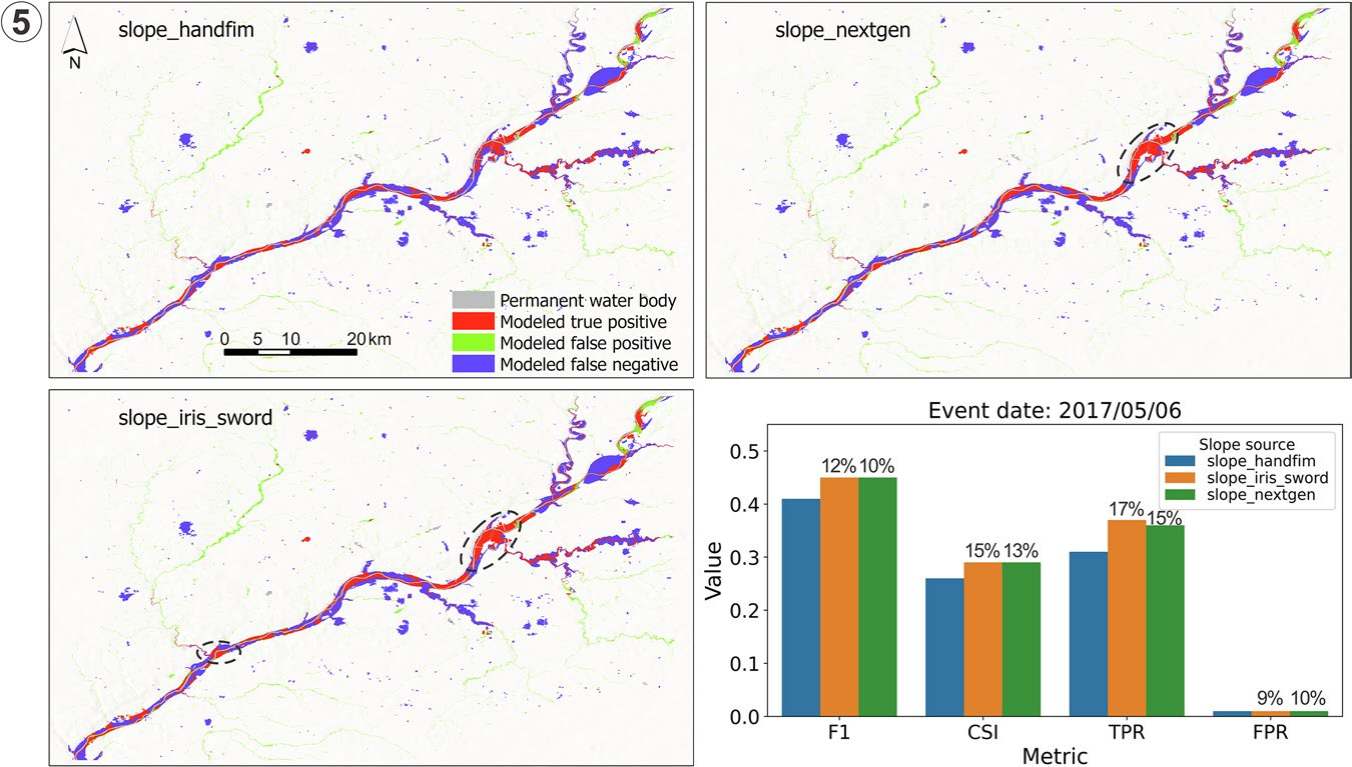
Comparison of flood maps based on OWP HAND-FIM using different slope sources and the evaluation results against the benchmark RS FIM for case event 5 (Fig. 9a). The dash-line circles highlight key FIM improvements relative to the original slopes. All slopes of *slope_iris_sword* are from IRIS.

**Fig. 12:**
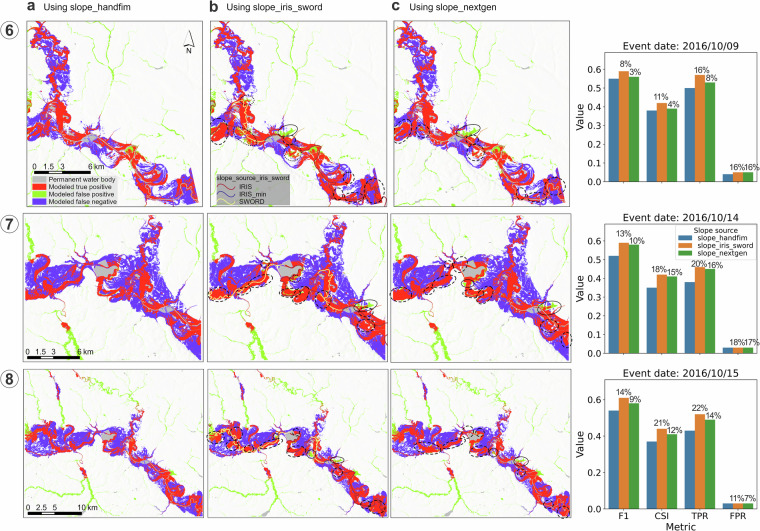
Comparison of flood maps based on OWP HAND-FIM using different slope sources and the evaluation results against the benchmark RS FIM of case events 6–8 (Fig. 9a). The dash-line circles indicate the locations where FIM is improved by *slope_iris_sword* or *slope_nextgen*, whereas solid-line circles indicate where overpredictions occur, and dot-line circles (three in 6-b and one in the middle of 7-b) indicate where improvements are dominant but with some overpredictions. The slope source IRIS_min marks locations with unavailable IRIS and zero SWORD slope in the FIM HF IRIS dataset, where the dataset’s minimum IRIS slope was used to avoid unrealistic discharge calculations in the OWP HAND-FIM.

The results show that *slope_iris_sword* significantly improved FIM accuracy across all locations and events (Fig. 9b), with average improvements of 24%, 31%, and 35% in F1 score, CSI, and TPR, respectively. Although the updated slopes led to a certain degree of overprediction (an average increase of 64% in FPR), this was mainly due to the originally very low overpredictions by *slope_handfim* (an average FPR of 0.02; Figs. 10–12). Overpredictions were still very low for the *slope_iris_sword* (on average 0.03 of FPR; Figs. 10–12), except for a few extreme cases.

To assess the accuracy of *slope_iris_sword* relative to the NextGen HF slopes (*slope_nextgen*), we generated FIM for the 8 case events using the NextGen HF slopes (Fig. 9 were similarly evaluated against the RS flood maps. The results show that *slope_nextgen* yielded an average improvement of 17% in F1, 22% in CSI, and 28% in TPR compared to *slope_handfim*. There was an average increase of 80% in FPR for the maps generated using *slope_nextgen* (Fig. 9c), but still with a low average FPR of 0.03 (Figs. 10–12). Overall, the improvements by *slope_nextgen* are lower than those by *slope_iris_sword*, and *slope_nextgen* also caused more overpredictions (Fig. 9). These indicate that *slope_iris_sword* has higher accuracy than the NextGen HF slopes. This may be because IRIS (v2.6) was derived using long-term aggregation (median) of recent observations between October 2018 and May 2024^43^, possibly more accurately reflecting the spatial variation of river slopes.

Further inspection of the overpredictions caused by the new slopes (*slope_iris_sword* and *slope_nextgen*) revealed the following:

1. In Figures 1–10, both SWORD (Figs. 1–13) and NextGen slopes resulted in large overprediction areas. This may reflect intrinsic limitations of the OWP HAND-FIM model^15^ and/or inadequate slope representation for these reaches in both datasets.

**Fig. 13:**
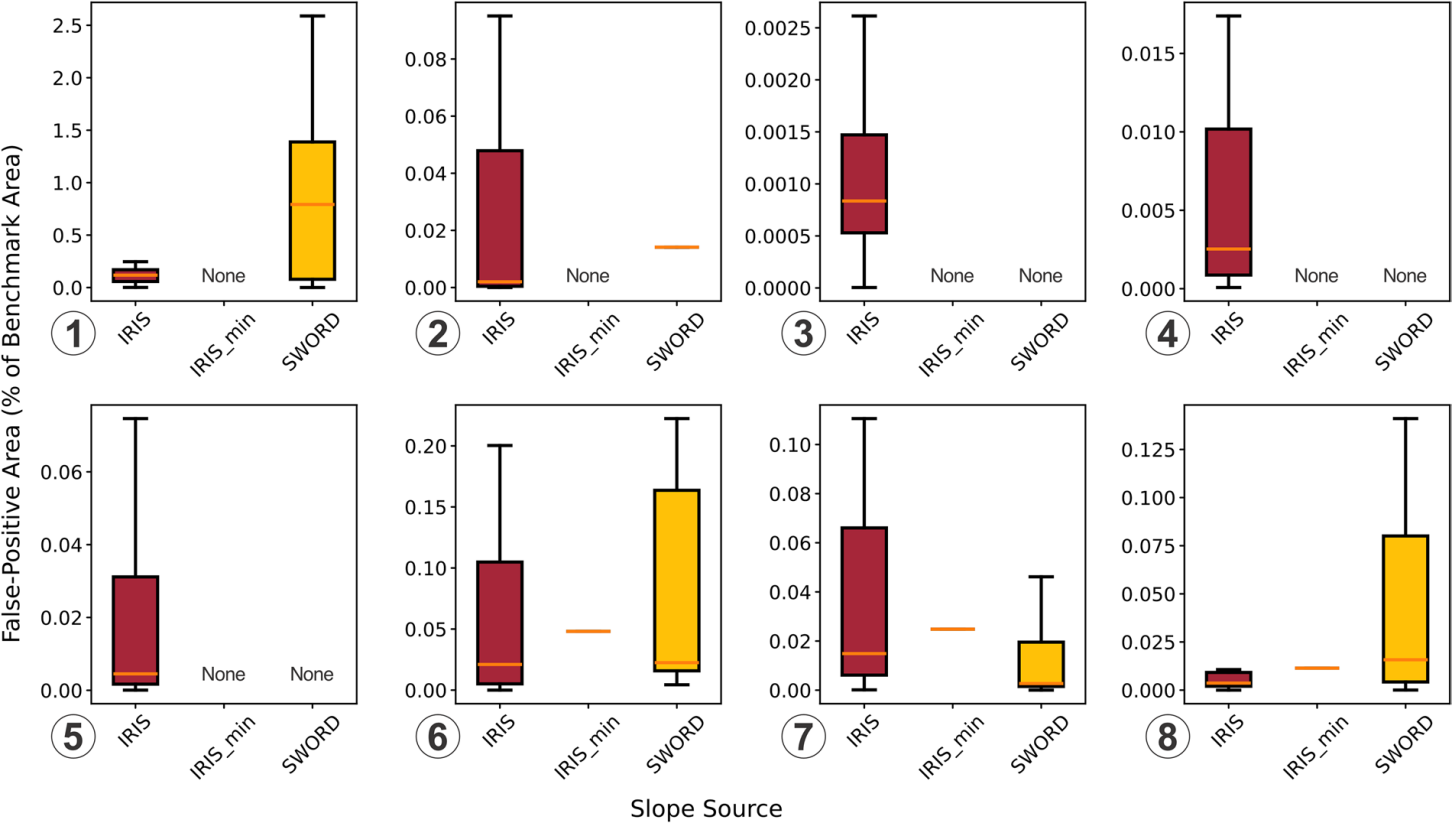
Percentage of false-positive FIM area associated with reach (via HAND catchment^15^), relative to the benchmark RS FIM area, grouped by slope source (*slope_source_iris_sword*) of the reach. Numbers in circles correspond to flood event numbers listed in Table 3. “None” indicates that the slope source was not used for any reach of that event.
2. In Fig. 12b, a major overprediction associated with an IRIS slope was observed across three flood events. This may be due to the coarse resolution of the corresponding IRIS (SWORD) reach, which spans 16.7 km, compared to the 1.3 km length of the associated FIM HF reach. Such a mismatch suggests that IRIS data may not adequately represent slope variability, particularly in meandering river segments^35^. This suggests that improving the resolution of river slope datasets could be beneficial, e.g. denser slopes can be calculated using water surface elevations between consecutive nodes (~200 m spacing) within the reach in the SWOT Vector Dataset^25,44^.
3. Similarly, the FIMs using *slope_nextgen* showed overpredictions at the same locations as those using IRIS slopes (Fig. 12c), indicating that NextGen may also fail to capture slopes in meandering reaches, or that the OWP HAND-FIM model itself may struggle with such locations.
4. Additionally, SWORD slopes led to noticeable overprediction in Event 8 (see solid-line circle in the middle of Figs. 8b–12), while not causing similar issues in Events 6 and 7 (Figs. 12b, 8–13). This points to possible sensitivity of the OWP HAND-FIM model to slope inputs, which warrants further investigation.

Overall, the magnitude of FIM overpredictions does not appear to differ significantly between IRIS and SWORD slopes in the tested events. Instead, the primary sources of error may stem from the OWP HAND-FIM model itself and/or inadequate slope representation of IRIS, SWORD, and NextGen, especially in meandering river sections.

**Fig. 14:**
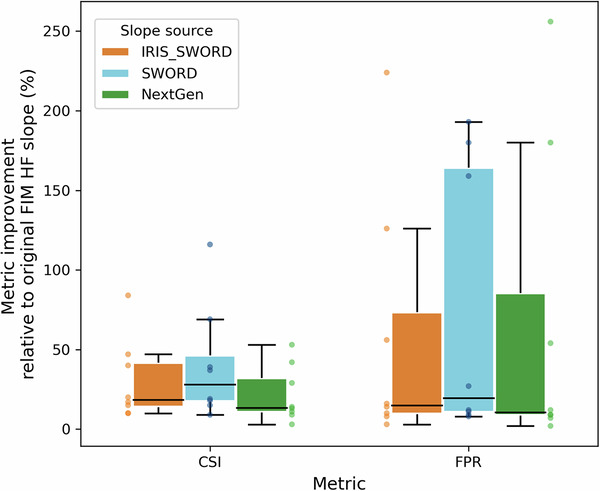
Evaluation metrics of FIMs using IRIS_SWORD slopes, all SWORD slopes, and NextGen slopes, relative to original FIM HF slopes, across eight test events (the points; Fig. 9a). CSI, critical success index; FPR, false positive rate.

To thoroughly evaluate the impact of DEM (MERIT Hydro)-derived SWORD slopes on FIM^25^, we generated FIMs using only SWORD slopes for the eight test events and compared them to remote sensing benchmark FIMs. Surprisingly, SWORD slopes outperformed both IRIS_SWORD slopes (where only a few reaches used SWORD, Figs. 10–12) and NextGen slopes, with an average CSI improvement of 40%, compared to 31% for IRIS_SWORD and 22% for NextGen (Fig. 14). However, SWORD slopes also led to higher false positive rates, while IRIS_SWORD showed more consistent improvements. Also given IRIS’s more recent observation period, long-term aggregation, and higher accuracy supported by previous studies^6,8,35^, we retained IRIS as the primary slope source for the FIM HF IRIS and NextGen HF IRIS datasets.

Though SWORD slopes produced higher and variable FPRs (Fig. 14), the average FPR remained low (0.04, vs. 0.03 for IRIS_SWORD), and overpredictions were generally comparable, except for one extreme case likely due to SWORD slopes and/or limitations in OWP HAND-FIM (Figures 1–10, 1–13). Also, only 13% of reaches in our dataset use SWORD slopes, and we clearly indicate slope sources (Table 2), allowing users to choose based on their preference. Overall, we believe that including SWORD slopes does not compromise dataset accuracy, and users can opt to use only IRIS slopes if desired.

## Usage Notes

The FIM HF IRIS dataset can be utilized by the OWP HAND-FIM model for flood inundation mapping by joining the *slope_iris_sword* to the OWP HAND-FIM HF using the common attribute *HydroID*, in combination with *From_Node* and *To_Node* (Tables 1, 2). We also provided a Python-based Jupyter notebook^46,47^ to facilitate the integration of the new river slopes into the OWP HAND-FIM model. Since this process involves several complex procedures, the notebook automates the re-generation of the hydrotable (containing SRCs and associated reach attributes) required for running the OWP HAND-FIM model to produce FIM^15^. Similarly, the NextGen HF IRIS dataset can be used by the NextGen HF, which supports various applications in modeling of hydrology, water resources, and flooding^17^. The integration involves joining the *slope_iris_sword* to the NextGen HF using the common attribute *id* (Table 1).

Note, as the SWORD dataset contains less flowlines than the FIM HF, and considering that NextGen HF has similar coverage of rivers as FIM HF^16,17^ and that NextGen HF slopes can improve FIM (Figs. 9c, 10–12), despite that they do not perform as well as the *slope_iris_sword*, the NextGen HF slopes can be a good alternative for the FIM HF reaches where IRIS slopes are unavailable.

FIM HF IRIS and NextGen HF IRIS will be updated regularly by spatially joining the routinely updated SWORD dataset^25,51^ (expected to further enhance the accuracy of SWORD flowlines and their topology) and the IRIS dataset^35,43^ (expected to incorporate future ICESat-2 observations). This can help to reduce the proportion of reaches relying on SWORD slopes, prone to overpredict the FIM, in the future update of our datasets. Additionally, both datasets include links to the latest SWOT Vector Data Product^25,44^, based on the common attribute *reach_id* (Table 1). This connection allows the SWOT Vector Data Product to be linked with the FIM HF and NextGen HF.

**Fig. 15:**
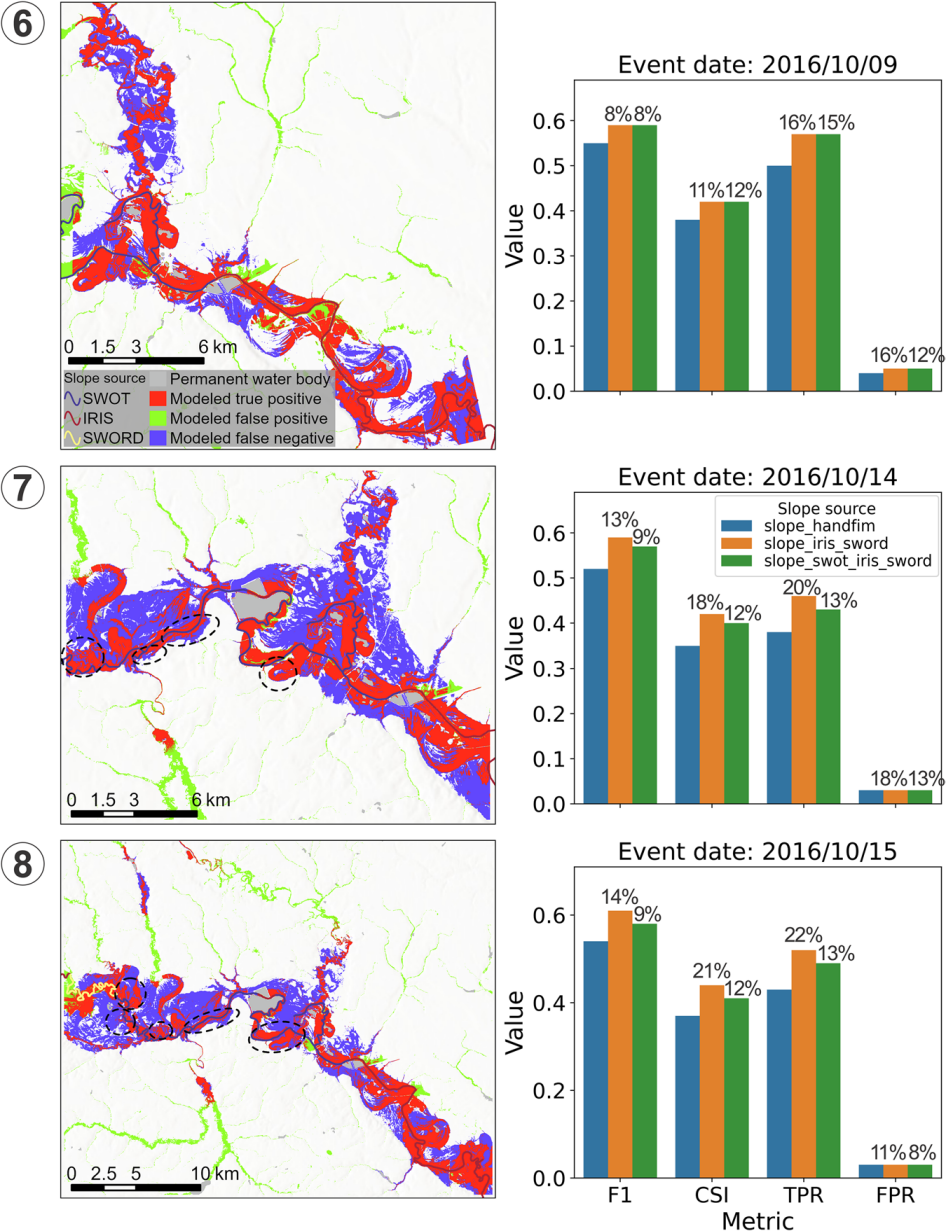
Assessments of flood inundation maps based on OWP HAND-FIM using SWOT slopes, through comparing with RS flood maps, for the case events 6–8 (Fig. 9a). For the reaches in which SWOT is unavailable, the *slope_iris_sword* was used, i.e. *slope_swot_iris_sword*. The slope source is indicated by reach colors. The dash-line circles indicate where flood maps using the original *slope_handfim* were improved by *slope_iris_sword* (Fig. 12b) but not by SWOT slopes.

The SWOT dataset is expected to provide river slopes with higher accuracy compared to IRIS^6,8^. To demonstrate the capacity of our datasets to link SWOT datasets and preliminarily test the potential of SWOT for improving FIM, we used the median river slopes from the SWOT Level 2 River Single-Pass Vector Reach Data Product, Version C (https://podaac.jpl.nasa.gov/dataset/SWOT_L2_HR_RiverSP_reach_2.0) collected between 2024/09/23 and 2024/11/04 to generate flood maps (Fig. 15). The results indicate that these short-term aggregated slopes improved FIM accuracy relative to the original FIM HF slopes, achieving average improvements of 8% in F1, 12% in CSI, and 14% in TPR. However, they did not perform as well as *slope_iris_sword* (Fig. 15). For certain reaches, SWOT slopes performed comparably and reduced overprediction compared to *slope_iris_sword* (Figs. 6–15). These findings suggest that SWOT river slopes have large potential to further improve FIM, also allowing the incorporation of time-varying (e.g. monthly and seasonal) and recently acquired slope observations.

## Data Availability

FIM HF IRIS, FIM HF NextGen, and the script for using FIM HF IRIS within the OWP HAND-FIM are available at Zenodo: https://zenodo.org/records/14885156 and HydroShare: 10.4211/hs.1532f4cb360244f9a6ba772ebd428180. CONUS-scale FIM HF flowlines (v4.5.2.11), originally downloaded from NOAA NWS OWP FIM AWS S3 bucket, are publicly accessible at CIROH AWS S3 bucket: https://ciroh-owp-hand-fim.ciroh.org/index.html. SWORD (v16) and IRIS (v2.6) are available at Zenodo with https://zenodo.org/records/10013982 and https://zenodo.org/records/13285066, respectively. NextGen HF (v2.2) is available at LynkerSpatial: https://www.lynker-spatial.com/data?path=hydrofabric%2Fv2.2%2F. NOAA Emergency Response Imagery: https://storms.ngs.noaa.gov/. GitHub Repository of the framework to spatially join FIM HF flowlines with SWORD flowlines: https://github.com/sdmlua/RiverJoin. GitHub Repository of FIMserv: https://github.com/sdmlua/fimserve. GitHub Repository of FIMeval: https://github.com/sdmlua/fimeval.
